# Total and high-molecular-weight adiponectin levels in relation to insulin resistance among overweight/obese adults

**DOI:** 10.5195/cajgh.2013.55

**Published:** 2013-10-04

**Authors:** Sushama D. Acharya, Rhobert W. Evans, Maria M. Brooks, Faina Linkov, Lora E. Burke

**Affiliations:** 1 School of Nursing, University of Pittsburgh; 2 Graduate School of Public Health, University of Pittsburgh; 3 School of Medicine, University of Pittsburgh

**Keywords:** Adiponectin, high molecular weight adiponectin, insulin resistance, homeostasis model assessment of insulin resistance (HOMA), weight loss and adiponectin levels

## Abstract

**Objective:**

To determine whether baseline levels or intervention-associated changes in total and high molecular weight (HMW) adiponectin levels were associated with insulin resistance after six months of behavioral treatment for weight loss.

**Design:**

An ancillary study to a behavioral weight loss trial; the intervention was delivered in group sessions.

**Methods:**

Participants included 143 overweight/obese adults with a mean BMI of 33.7 kg/m^2^. The sample was 88% female, 67% white, and 44.2 ± 8.5 years old. Circulating adiponectin levels (total and HMW) and the homeostasis model assessment (HOMA) of insulin resistance were measured and evaluated.

**Results:**

At baseline, there was significant inverse associations between total adiponectin and HOMA (*p* < 0.001) and between HMW adiponectin and HOMA (*p* < 0.001) independent of weight. At 6-mo, there was a 17% improvement in HOMA, 8% increase in total adiponectin, 17% increase in HMW adiponectin levels, and 8.72% weight loss (*p’*s for all< 0.001). There was also a significant inverse association between changes in total adiponectin and HOMA (*p* = 0.04) that was independent of baseline weight and weight loss. In contrast, the association between changes in HMW adiponectin and HOMA was attenuated after adjustment for weight loss.

**Conclusions:**

An increased level of total adiponectin was associated with improved insulin sensitivity, regardless of baseline weight and weight loss. However, baseline total and HMW adiponectin levels were more strongly associated with HOMA than changes in these measures at six months. HMW adiponectin level was not related more closely to insulin resistance than total adiponectin level.

Obesity is a significant public health problem with recent data indicating more than 1.4 billion overweight adults worldwide and nearly two-thirds of the global population living in countries where overweight and obesity affect mortality more than underweight.^1^ As obesity is a major risk factor for the development of insulin resistance (IR) and associated metabolic diseases including hypertension, hyperlipidemia, atherosclerosis, and certain types of cancer, significant research has been conducted to understand obesity mechanisms related to pathogens of these diseases. It is now evident that adipose tissue is no longer considered an inert tissue mainly devoted to energy storage but is now recognized as an active endocrine organ secreting several hormones and a diverse range of other protein factors.^2^ Adiponectin is one of the many hormones secreted by adipose tissue as the adipokine in the human body. IR and obesity are both associated with lower plasma adiponectin concentrations suggesting its important physiological role. 3 Improvements in IR, glucose uptake in skeletal muscles and hepatic fatty acid oxidation upon administration of adiponectin in animal studies suggest that adiponectin may provide a link between adiposity, IR and diabetes. 4 Figure 1 depicts a proposed mechanism linking adiposity and adiponectin with IR and diabetes.

A strong inverse relationship between adiponectin and IR has been reported in population cross-sectional studies. 5–7 Moreover, longitudinal data also suggest a relationship between lower adiponectin levels and the development of IR and diabetes. In a study of Pima Indians, those with high adiponectin concentration were found to be at a lower risk of developing diabetes than those with a low concentration, suggesting a potential role of adiponectin in the pathogenesis of diabetes. 8 Similarly, an inverse association between adiponectin and diabetes risk was reported among a large population of healthy women, 9 a middle-aged population cohort, 10 and adults with insulin-resistance. 11 Mather *et al.* reported baseline adiponectin as a better marker of diabetes prevention than changes in adiponectin levels over one year in the Diabetes Prevention Program (DPP). 12 However, no association was found between adiponectin and IR despite improved insulin sensitivity in response to weight loss among obese women, 13 obese adults, 14 and insulin resistant adults who were not diabetic. 15 Additionally, part of the difficulty also is that despite short term weight loss, successful long-term weight loss maintenance is extremely difficult as most individuals regain most of their weight with a few years.^16^ Because of these variations due to population, sample size, differences in baseline degree of insulin resistance, and adiponectin, the evidence provided by these studies on the relationship between IR and adiponectin in the presence of weight loss remains equivocal.

Adiponectin exists in circulation in three distinct forms: low molecular weight (LMW), medium molecular weight (MMW), and high molecular weight (HMW) forms.^3^,^17^ HMW adiponectin has been suggested to be the most active form of adiponectin and more closely associated with IR with an ability to enhance insulin action.^18^,^19^ However, a major limitation of many epidemiological and intervention studies is the measurement of only total adiponectin level. Thus, little is known about how the relationship between HMW adiponectin and IR might differ from the relationship between total adiponectin and IR. Hence, the aims of this study were to determine whether baseline levels or intervention-associated changes in total and HMW adiponectin levels were associated with IR after 6 months of behavioral treatment for weight loss.

## Materials and Methods

### Study design

**Figure 2:**
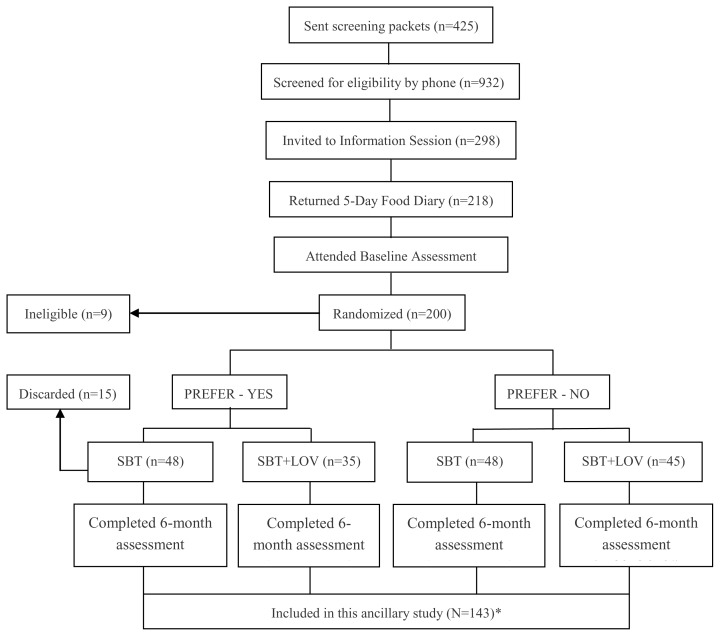
PREFER trial flowchart (included only 6-mo assessment). STD-D = Standard Diet; LOV-D = Lacto-Ovo-Vegetarian Diet *Blood samples on 94.7% of participants who attended the 6-mo assessment were stored and analyzed

This was an ancillary study to the PREFER trial, an 18-month behavioral weight loss study designed to evaluate the effects of treatment preference (Preference-Yes vs. Preference-No) and two dietary treatment options, standard calorie restricted low fat diet (STD-D) vs. lacto-ovo-vegetarian diet (LOV-D). Participants were randomly assigned first to one of the two preference conditions (yes or no). If assigned to the Preference-No condition, they were further randomly assigned to one of the two diet conditions, STD-D or LOV-D. If assigned to Preference-Yes condition, they were assigned to the diet they indicated as preferred at screening. The design, recruitment, and randomization procedures for the PREFER trial have been described in detail elsewhere. 20 Refer to Figure 2 for the number of participants at each stage. The study protocol was approved by the University of Pittsburgh Institutional Review Board; all participants provided written informed consent. The PREFER trial was conducted between 2000 and 2005, and unthawed aliquots of serum samples were stored at −80°C. The current analysis was limited to the 143 participants whose sera samples were available from baseline and 6-month assessments.

### Participants

The study population included adults between 18 and 55 years of age, a body mass index (BMI) between 27 and 43 kg/m^2^ inclusively, and adequately completed a 5-day food diary at screening. Individuals were excluded if they had diabetes or a medical condition requiring physician supervision of diet or physical activity, were pregnant, participated in a behavioral or pharmacological weight-loss program in the last 6 months, reported alcohol intake of ≥ 4 drinks per day, and reported abstention from eating meat, poultry, or fish in the past month.

### Intervention

All four treatment groups received the same standard behavioral intervention. The only difference between the diet groups was that the LOV-D participants were instructed to eliminate meat, poultry, and fish form their diet by the 6th week of the program. Details of the intervention have been reported elsewhere. 20 In brief, all participants received a daily energy and fat gram goal based on gender and baseline body weight as described in Table 1. Participants were instructed to increase their physical activity gradually, primarily via walking, until they reached a goal of 150 minutes by week 6. As an alternative to walking, aerobic activities such as bicycling, swimming, and jogging were encouraged. Use of frequent, short bouts to meet one’s exercise goal, for example exercising for 10–15 minutes three times per day, was also promoted. The intervention group sessions were held weekly during the first six months. The cognitive-behavioral intervention used several strategies from models of motivation and behavior change. All participants received nutritional and behavioral counseling and were provided with practical hands-on experience to develop skills to implement a healthy lifestyle. All participants self-monitored their daily energy and fat intake, as well as physical activity (duration and type) they performed during the study period.

### Measures

Baseline demographic characteristics were collected via a self-administered, standardized questionnaire. All anthropometric, biochemical, and adiponectin measurements were obtained at baseline and 6 months. Weight was measured on a digital scale (Tanita Corporation of America, Inc., IL) in light clothing and without shoes. Height was measured with a wall-mounted stadiometer. The physical activity assessment was conducted via a self-administered Paffenbarger Activity Questionnaire.^21^ This questionnaire has been shown to have good test-retest reliability. A metabolic equivalent value was assigned to each leisure activity, from which the total energy expenditure was calculated. Blood samples, obtained following a 12-hour fast were assayed at the Heinz Nutrition Laboratory, University of Pittsburgh. Plasma glucose was measured with the use of the hexokinase-glucose 6-phosphate dehydrogenase enzymatic assay (Sigma Diagnostics, St. Louis, MO), and insulin concentration was measured by a radioimmunoassay kit (Linco Research, St. Charles, MO). Insulin resistance was assessed by homeostasis model assessment of insulin resistance (HOMA-IR) and was calculated as fasting insulin concentration (U/mL) x fasting glucose concentration (mmol/L)/22.5. HOMA is a mathematical assessment of the balance between hepatic glucose output and insulin secretion and has been widely validated and applied for quantifying insulin resistance and β-cell function. Serum levels of total and HMW adiponectin were determined using the ELISA technique (ALPCO Diagnostics, Salem, NH). The intra-assay and inter-assay CVs were 6.4% and 12.7% for total adiponectin and 6.4% and 12.6% for HMW adiponectin, respectively. All samples were assayed in duplicate.

### Statistical analysis

Statistical analyses were performed using SAS (version 9.2; SAS Institute Inc, Cary, NC). All continuous variables were checked for normality. Basic statistics were expressed as mean ± SD or as proportions, unless otherwise specified. For all continuous variables, comparisons between baseline and 6-month measures were examined using a paired sample’s t-test. A chi-square test was performed for comparing the categorical variables at baseline. Associations between continuous variables were assessed using the Pearson or Spearman correlation coefficient. Separate multiple linear regression models were used for HOMA at baseline and change in HOMA score (6-month – baseline scores) as the dependent variables to examine associations between total and HMW adiponectin at baseline and 6 months. The models were adjusted for age, gender, race, baseline weight, baseline energy expenditure, changes in weight, and energy expenditure. All tests performed were two-sided, and significance level was set at *p* < 0.05.

## Results

Of the 176 participants randomized at baseline, 151 completed the 6-month assessment, and the sera samples of 143 (95%) participants were available from both baseline and 6-month assessments. The majority of participants were female (88%), white (67%), currently married or living with a partner (65%), and employed (94%). The mean age was 44.2 ± 8.5 years old with a BMI range of 26.71 to 42.56 kg/m^2^ (Mean= 33.78 kg/m^2^) and, on average, 15.3 years of formal education. No differences in baseline characteristics were found between participants who were excluded and those included in the analysis. There was no significant difference in HOMA between the two diet groups; STD-D vs. LOV-D (4.28 ± 2.15 vs. 4.62 ± 2.32; *p* = 0.3), nor were there differences by the two preference groups; Preference-Yes vs. Preference-No (4.60 ± 2.18 vs. 4.28 ± 2.27; *p* = 0.4). Additionally, there were no significant differences in total adiponectin level by diet group (7.82 ± 3.74 vs. 7.64 ± 2.87 ug/ml; *p* = 0.7) or preference group (7.76 ± 3.57 vs. 7.62 ± 7.66 ug/ml; *p* = 0.7), nor did HMW adiponectin level differ by diet group (3.33 ± 2.21 vs. 3.23 ± 1.68 ug/ml; *p* = 0.8) or preference group (3.30 ± 1.97 vs. 3.23 ± 20.01; *p* = 0.8). Since there were no significant differences between the two diet groups and the two preference groups, further analyses were conducted as a single sample without regard to randomized diet or preference condition.

**Figure 3:**
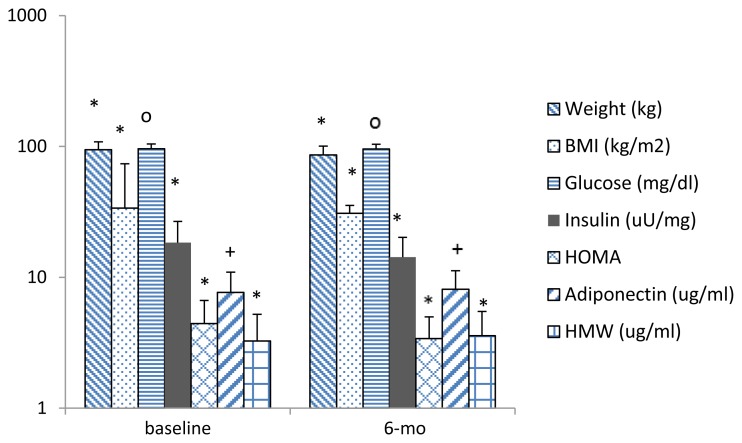
Measures at baseline and 6-month assessments.

At baseline, HOMA did not differ by gender (female vs. male: 4.36 ± 2.23 vs. 4.97 ± 2.24; *p* = 0.3) or race (white vs. non-white: 4.47 ± 2.34 vs. 4.33 ± 20.00; *p* = 0.7). However, females had significantly higher total (7.85 ± 3.32 vs. 6.31 ± 2.58 ug/ml; *p =* 0.04) and HMW adiponectin levels (3.37 ± 2.9 vs. 2.39 ± 1.43 ug/ml; *p =* 0.03) than males. Figure 3 describes the baseline and 6-month measures of the studied variables. From baseline to 6 months, there was a 17% reduction in HOMA (*p* < 0.001), 8% increase in total adiponectin (*p* = 0.001), and 17% increase in HMW adiponectin levels (*p* < 0.001). Further, there was a significant weight loss (8.72%) at 6 months (*p* < 0.001). There were highly significant correlations between total and HMW adiponectin levels at baseline (r = 0.95; *p* < 0.001) and also at 6 months (r = 0.95; *p* < 0.001). At baseline and 6 months, weight, BMI, glucose, and insulin showed significant positive associations with the HOMA score while total and HMW adiponectin levels showed significant negative association with HOMA (*p* for all < 0.001).

Separate multiple regression analyses were performed for both baseline and 6-month measures with baseline HOMA and the change in HOMA scores as the dependent variables, respectively. The final model, with baseline measures adjusted for weight, indicated significant inverse associations between total adiponectin and the HOMA score (*p* < 0.001) and between HMW adiponectin and HOMA (*p* < 0.001). The estimates for total and HMW adiponectin still remained highly significant after adjustment for baseline weight (Table 2). At 6 months, the final regression model revealed a significant inverse association between changes in total adiponectin and HOMA (*p* = 0.04) that was independent of baseline weight and weight loss. In contrast, higher HMW adiponectin was associated with improvements in the HOMA score without weight loss in the model (*p* = 0.02), but the association was no longer significant after adjustment for weight loss (Table 3). Additional adjustment for energy expenditure at baseline and 6 months did not alter the results.

## Discussion

A better understanding of the extent of the association of adiponectin with IR in obesity might help clarify mechanisms of insulin sensitivity, which in turn, might be beneficial in identifying preventive approaches to enhance insulin sensitivity and thereby reduce the risk for developing diabetes. The study’s findings revealed that the higher baseline levels of total and HMW adiponectin levels were significantly associated with lower HOMA score, independent of body weight. At 6 months there were significant reductions in the HOMA score and weight, which paralleled the improvements in total and HMW adiponectin levels. The increase in total adiponectin was inversely associated with a decrease in the HOMA score independent of baseline weight and weight change. However, the significant association between changes in HMW adiponectin level and the HOMA score was attenuated after adjustment for weight change.

There were significant inverse associations of total and HMW adiponectin levels with the HOMA score at baseline, even after adjusting for baseline weight, indicating an independent association of total and HMW adiponectin levels with the HOMA score regardless of the degree of obesity. The association between total adiponectin and HOMA is consistent with findings from previous studies among individuals with type 2 diabetes, 22 a representative community adult population, 6 and a community-based cohort with and without the metabolic syndrome. 5 The DPP study also reported an inverse association between baseline total adiponectin levels and future diabetes independent of baseline adiposity among individuals at increased risk for diabetes. 12 The findings suggest the persistence of an independent role of not only total adiponectin but also HMW adiponectin levels in relation to IR among a generally healthy overweight/obese population with no diabetes or clinical vascular disease.

The association between changes in total and HMW adiponectin levels and HOMA scores were examined separately after 6 months of intervention. The degree of univariate association between HOMA and total and HMW adiponectin levels at both baseline and 6 months remained unchanged. There were intervention-associated significant reductions in weight, the HOMA score and improvements in total, and HMW adiponectin levels. The increase in total and HMW adiponectin was associated with the change in weight suggesting that change in total and HMW adiponectin levels might reflect body weight change. The multiple regression analysis showed that an increased level of total adiponectin contributed to improved insulin sensitivity independent of baseline weight and change in weight. Although the estimate for change in total adiponectin decreased by 34% when change in weight was added to the model, these results indicate that improvements in total adiponectin level contributed to improved insulin sensitivity above the usual clinical markers of diabetes. The present study highlights the importance of adiponectin in the development of IR and subsequent development of Type 2 diabetes. Thus, interventions, such as the one used in this study, that target weight loss and increase total adiponectin levels may improve insulin sensitivity and reduce the risk of diabetes for overweight individuals.

A few studies have reported HMW adiponectin to have a stronger association than total adiponectin level in the incidence of type 2 diabetes^23^,^24^ and insulin sensitivity.^17^–^19^,^25^ Most of these studies either had only a single measurement of HMW adiponectin level,^19^,^23^–^25^ were limited by the small sample size, 15,17,18 or had a pharmacological intervention. 26 There was a highly significant association between total and HMW at baseline. Bluher *et al.* reported no superiority of HMW adiponectin over total adiponectin values in predicting insulin sensitivity. 27 Likewise, the study results also showed a similar magnitude of correlations between both total and HMW adiponectin levels with the HOMA score at baseline suggesting no clinical differences between total and HMW adiponectin levels in relation to insulin resistance.

At 6 months, the significant association between changes in HMW adiponectin level and HOMA was attenuated after adjustment for change in weight and was no longer significant, despite an increase in HMW adiponectin that was more than double the increase in total adiponectin. This finding indicates a possible threshold level of HMW adiponectin that might be needed to have an independent effect on change in the HOMA score. Alternatively, a different pathway of an intervention-associated change in HMW adiponectin level from the baseline HMW adiponectin level could not be ruled out as a possibility. Regardless of the mechanism, the study refutes the hypothesis of a predominant role of HMW adiponectin level over total adiponectin level in relation to IR. The study also provided a unique opportunity to examine if baseline or intervention associated changes in total and HMW adiponectin levels were more strongly associated with IR. The degree of association between total and HMW adiponectin level were stronger at baseline than the relationship between changes in total adiponectin and the HOMA score at 6 months. Moreover, the significant association between change in HMW adiponectin level and HOMA score was taken over by weight loss. Consistent with the findings of Mather *et al*., 12 baseline total and HMW adiponectin levels were more closely related to IR than the changes in these measures at 6 months.

The potential mechanism of how plasma adiponectin levels influence IR is still not known. A higher level of adiponectin level has been reported to increase fatty acid oxidation with subsequent reduction of triglycerides thereby directly sensitizing the body to insulin and reserving IR in animal models of obesity and diabetes 28 and in humans. 25 Through in vitro studies, adiponectin has been shown to trigger the protein kinase, an insulin independent enzyme known to stimulate glucose use and increase fatty acid oxidation in skeletal muscle. 4 It has been reported that some inflammatory markers, such as tumor necrosis factor-α, interleukin-6 or C-reactive protein have an adiponectin inhibitory effect, which in turn may lead to IR.^7^,^29^ It is possible that the levels of these markers decrease with the corresponding increase in adiponectin levels with weight loss and may improve insulin sensitivity.

A main limitation of the study included a measure of IR with a surrogate marker. However, HOMA derived from the mathematical model has been shown to be an adequate indicator of IR and has been used in many epidemiological and clinical studies. Also, the generalizability of these findings may be limited due to the relatively homogenous study population; however, the minority representation exceeded that of the local community. The strengths of the study included the measurements of both total and HMW adiponectin. An additional strength was its longitudinal design and examination of the relationship between total and HMW adiponectin levels with IR cross-sectionally and over time. Moreover, the clinical measures were obtained via a standardized protocol and assays were performed with good precision and the study included a larger sample than what has been reported in the literature.

## Conclusions

An increased level of total adiponectin contributed to improved insulin sensitivity regardless of baseline weight and weight loss. However, the association of change in total adiponectin and HOMA score was modest compared to baseline measures suggesting that the cross-sectional measure of total adiponectin might be a comparable indicator of IR and subsequent risk of developing diabetes among overweight or obese adults. These findings provide support for the importance of adiponectin levels and a need for their improvements to prevent diabetes. Thus, interventions that enhance adiponectin secretion or action may have potential for diabetes risk reduction. Improvements in the HMW adiponectin level were largely explained by the weight loss suggesting the role of HMW adiponectin level is no more important than that of total adiponectin in relation to improved insulin sensitivity. These findings provide evidence for the importance of weight loss as a significant public health preventive measure to enhance adiponectin levels among the studied population, which could impact the progression of atherosclerosis and associated metabolic diseases.

## Figures and Tables

**Figure 1:**
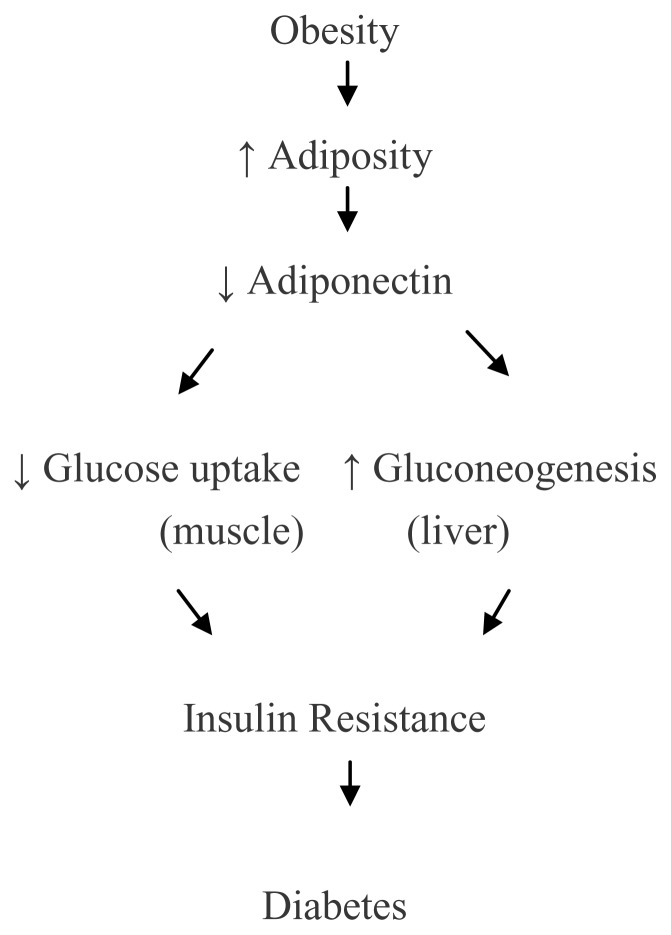
Proposed link among adiposity, adiponectin, insulin resistance and diabetes.

**Table 1:** Daily dietary goals.

Baseline weight (lbs)	Prescribed daily goals			

	Energy intake (kcal)		Total fat intake (g)*	

	Male	Female	Male	Female
< 200	1500	1200	42	33
≥ 200	1800	1500	50	42

* 25% of the total energy intake for everyone.

**Table 2:** Multiple regression models of total and HMW adiponectin levels on baseline HOMA measure (N = 143).

Models	Baseline HOMA			R^2^	Models	Baseline HOMA			R^2^
	
	Estimate	s.e.	*P*			Estimate	s.e.	*P*	
Model 1					Model 1				
Adiponectin (ug/ml)	−0.28	0.05	< 0.001	0.17	HMW (ug/ml)	−0.42	0.09	< 0.001	0.14
Model 2					Model 2				
Adiponectin (ug/ml)	−0.29	0.05	< 0.001	0.18	HMW (ug/ml)	−0.45	0.09	< 0.001	0.15
Gender	−0.12	0.55	0.83		Gender	−0.10	0.56	0.85	
Race	−0.40	0.37	0.28		Race	−0.52	0.38	0.17	
Age (yr)	−0.02	0.02	0.27		Age (yr)	0.01	0.02	0.40	
Model 3					Model 3				
Adiponectin (ug/ml)	−0.26	0.05	< 0.001	0.30	HMW (ug/ml)	−0.38	0.09	< 0.001	0.27
Gender	0.45	0.34	0.39		Gender	0.44	0.54	0.42	
Race	−0.46	0.01	0.18		Race	0.55	0.36	0.12	
Age (yr)	0.02	0.02	0.20		Age (yr)	0.02	0.02	0.32	
Weight (kg)	0.06	0.01	< 0.001		Weight (kg)	0.06	0.01	< 0.001	

HMW= high molecular weight; HOMA= homoeostasis model assessment of insulin resistance.

Reference groups: gender-males, race- white

**Table 3:** Multiple regression models of total and HMW adiponectin levels on change in HOMA measure at 6 months (N = 143).

Models	Change in HOMA			R^2^	Models	Change in HOMA			R^2^
	
	Estimate	s.e.	*P*			Estimate	s.e.	*P*	
Model 1					Model 1				
Adiponectin Δ (ug/ml)	−0.25	0.09	0.004	0.06	HMW Δ (ug/ml)	−0.34	0.14	0.02	0.04
Model 2					Model 2				
Adiponectin Δ (ug/ml)	−0.25	0.08	0.005	0.11	HMW Δ (ug/ml)	−0.32	0.15	0.03	0.09
Gender	0.95	0.43	0.03		Gender	0.99	0.43	0.02	
Race	0.38	0.29	0.20		Race	0.33	0.30	0.28	
Age (yr)	0.01	0.01	0.9		Age (yr)	0.0003	0.01	0.98	
Model 3					Model 3				
Adiponectin Δ (ug/ml)	−0.26	0.09	0.003	0.16	HMW Δ (ug/ml)	−0.34	0.15	0.02	0.14
Gender	0.64	0.43	0.14		Gender	0.68	0.44	0.12	
Race	0.44	0.29	0.13		Race	0.38	0.29	0.19	
Age (yr)	−0.000	0.02	0.97		Age (yr)	−0.001	0.02	0.92	
Baseline weight (kg)	−0.03	0.009	0.003		Baseline weight (kg)	−0.03	0.009	0.003	
Model 4					Model 4				
Adiponectin Δ (ug/ml)	−0.17	0.08	0.04	0.27	HMW Δ (ug/ml)	−0.18	0.14	0.21	0.26
Gender	0.06	0.42	0.88		Gender	0.10	0.48	0.82	
Race	0.46	0.27	0.09		Race	0.40	0.27	0.14	
Age (yr)	0.0005	0.01	0.97		Age (yr)	0.000	0.01	0.99	
Baseline weight (kg)	−0.03	0.009	0.005		Baseline weight (kg)	−0.02	0.008	0.006	
Weight Δ (kg)	0.10	0.02	< 0.0001		Weight Δ (kg)	0.10	0.02	< 0.0001	

HMW=high molecular weight; HOMA= homoeostasis model assessment of insulin resistance; Δ=change.

Changes values were defined as 6 months - baseline.

Reference groups: gender-males, race- white
